# Developing a kidney and urinary pathway knowledge base

**DOI:** 10.1186/2041-1480-2-S2-S7

**Published:** 2011-05-17

**Authors:** Simon Jupp, Julie Klein, Joost Schanstra, Robert Stevens

**Affiliations:** 1 School of Computer Science, University of Manchester, UK; 2 Institut National de la Santé et de la Recherche Médicale (INSERM), U858, Toulouse, France; 3 Université Toulouse III Paul-Sabatier, I2MR, IFR150, Toulouse, France

## Abstract

**Background:**

Chronic renal disease is a global health problem. The identification of suitable biomarkers could facilitate early detection and diagnosis and allow better understanding of the underlying pathology. One of the challenges in meeting this goal is the necessary integration of experimental results from multiple biological levels for further analysis by data mining. Data integration in the life science is still a struggle, and many groups are looking to the benefits promised by the Semantic Web for data integration.

**Results:**

We present a Semantic Web approach to developing a knowledge base that integrates data from high-throughput experiments on kidney and urine. A specialised KUP ontology is used to tie the various layers together, whilst background knowledge from external databases is incorporated by conversion into RDF. Using SPARQL as a query mechanism, we are able to query for proteins expressed in urine and place these back into the context of genes expressed in regions of the kidney.

**Conclusions:**

The KUPKB gives KUP biologists the means to ask queries across many resources in order to aggregate knowledge that is necessary for answering biological questions. The Semantic Web technologies we use, together with the background knowledge from the domain’s ontologies, allows both rapid conversion and integration of this knowledge base. The KUPKB is still relatively small, but questions remain about scalability, maintenance and availability of the knowledge itself.

**Availability:**

The KUPKB may be accessed via http://www.e-lico.eu/kupkb.

## Introduction

The early detection and better understanding of (chronic) renal disease is important as it will reach pandemic proportions over the next few decades [1]. The biologist’s goal in renal disease is to understand the pathological processes and identify disease biomarkers. This requires the analyses of experimental data from multiple biological levels (e.g. genes, proteins and metabolites). These data need to be integrated with existing knowledge from databases and the scientific literature to connect the different levels. In addition, the kidney field is peculiar for at least two reasons:

1. the kidney is highly cellular and compartmentalised and each compartment is involved in many different functions and,

2. most of the large scale data comes from analysis of urine, that needs to be put into the ‘kidney’ context.

All together this makes the analysis of data, for which integration of data is a pre-requisite, problematic. This paper presents a case-study for developing a knowledge base around a focused domain in the life sciences, namely the kidney and urinary pathway (KUP). The KUP Knowledge Base (KUPKB) is being developed as part of the e-LICO project [2]. e-LICO is developing a data mining platform that supports the semi-automated construction of data mining workflows for data intensive sciences [3]. The e-LICO platform is to be demonstrated with a system biology use case that uses real data encountered in the KUP domain. The data spans multiple -omic levels and is collected from different tissues and from different species. For example, most of the human -omics data originates from urine [4] and needs to be related back to the kidney and its parts. In contrast, multilevel -omics data from animal models is more regularly available. e-LICO aims to develop tools that will mine these large scale disparate experimental findings, link those to existing data and build new predictive models for renal disease.

The KUPKB is built using a Semantic Web approach in order to assess the benefits and feasibility of creating such a resource with this technology. The methodology section guides the reader through the creation of a Kidney and Urinary Pathway Ontology (KUPO), that provides a specialised application ontology for the KUP domain. The KUPO provides the schema for the data held in the KUPKB. Within this methodology we explore the requirements for tools that help engage the biologists in the design and construction of such an ontology. The results section describes the KUPKB with examples of the kinds of queries that can be asked across multiple biological levels. We conclude by discussing the merits and limitations of our approach.

## Background

Data integration in the life science is an ongoing challenge in Bioinformatics; problems arise because standards for data formats, identifiers, common vocabularies and agreed semantics between databases are lacking [5,6]. Data in the life sciences are complex and volatile that, when taken with the issues outlined, makes the necessary integration of life sciences data hard work. Another factor is the numerous data resources published by independent groups that leads to an expansion of the heterogeneities that are rife in life science data [7].

Developing new resources that integrate existing data typically involves centralising the external data within new bespoke schemas. This ‘warehousing’ approach is common in the life sciences and over time leads to an increasing number of resources, each with their own schema [7]. The situation with respect to accessing these data is, however, improving with data providers often offering programmatic access to the data via Web Services or database exports [8,9]. This access affords easier integration opportunities, despite the semantic heterogeneities and the problem of identity of entities within life science’s data. The *‘identity crisis*’ [10] is being addressed through efforts such as shared names [11] and services such as BridgeDB [12], but wide spread compliance has yet to be realised. The adoption of ontologies for the annotation of data is providing new possibilities for data integration that go beyond using primary database entry identifiers alone.

The problem is exacerbated in the life sciences due to the nature of the data being captured. Biological data are complex, heavily inter-related and also often irregular or incomplete [6,13-15]. Relational databases are good in situations where the data are regular and complete, and thus are not always suitable for life science data [16-18]. Ontologies offer a potential solution to this problem as they have been demonstrated to be good at modelling things that are irregular and incomplete [19,20]. Ontologies are designed to be extensible and can be used to build a conceptualisation of a domain. An ontology language, such as the Web Ontology Language (OWL) [21], provides a precise semantics for the language that can be used to check for consistency in data, along with querying and inference over data. Ontologies have become popular in the life sciences [22] for the annotation of data and offer novel ways for the analysis and integration of biological data. Despite the uptake of ontologies for annotating data, these annotations are often held in more traditional database systems that lack the necessary support to fully exploit the benefits an ontology brings.

The Semantic Web encompasses many ideas and technologies, but at its heart is the creation of a connected Web of semantically described data. As opposed to the existing Web of connected documents, the Semantic Web provides a framework to publish statements about entities in those documents [23]. At the core of the Semantic Web is the Resource Description Framework (RDF) [24] that uses Uniform Resource Identifiers (URI) to identify objects on the Web. The RDF data model provides a mechanism to make statements about these objects in the form of Subject, Predicate and Objects; these statements are commonly referred to as triples. This simple triple model can be used to create large connected graphs of data and expose them to application over the Web. The use of URIs and RDF to expose data on the Web enables the syntactic integration of data held in biological databases; this model is easily extensible by the addition of new triples and is not constrained to a particular schema. This light, syntactic reconciliation means that publishing data in RDF is easy. This ease is at least in part due to the lack of a schema. Using these data, however, can be hard due to the lack of a schema; this means the heterogeneity in the data still exist and, while a common syntactic form allows queries across different data represented as RDF, the query formulator still needs to reconcile the naming and conceptualisation of those data in order to formulate that query.

RDF alone provides little in the way of semantics for what these objects are and what the relationships mean. It does, however, offer a means of layering semantic information over its simple data model and data published according to that model. At their heart, ontologies provide a simple service by defining the entities as they appear in a domain’s information. ‘Knowing what there is’ and adopting a common means of naming and inter-relating ‘what there is’ offers a means of providing a semantic layer over the syntactic integration of RDF. A common understanding of the types of entities and the types of relationships between them provides the means to query those data; that is, ontologies offer a schema-like mechanism for RDF data. The Web Ontology Language (OWL) provides us with an ontology language that can be expressed using RDF. OWL ontologies can add semantics to data captured in RDF, these semantics facilitate a common model for the data, inference and expressive queries over the data.

The work presented in this paper builds on previous efforts to expose life science data on the Semantic Web. Ruttenburg, Antezana and Cheung give a wider overview of how Semantic Web technologies are being deployed in the life sciences [25-27]. Dhanapalan, Sahoo, Hugo and Antezana provide case studies from different domains for data integration on the Semantic Web [28-31].

Several public databases have been made available as RDF, the Bio2RDF project provides a repository of over forty biological database already available to download as RDF [32]. The W3C health care and life sciences working group (HCLS) provide guidelines and a case-study on Alzheimer’s Disease that was built on Semantic Web technology [33].

The KUPKB sits within this tradition. The KUPKB is distinguished by its means of production; we use a series of existing ontologies with a light-weight mechanism for integrating these ontologies to give a backbone of background knowledge for the KUP domain in the form of the KUPO. This ‘schema’ is populated with workflows that bring in the various pre-existing KUP resources.

## Methodology

An initial set of experiments were chosen by the KUP scientists for inclusion in the KUPKB. These experiments span over different biological levels (genes or proteins), different techniques, different species (mouse or human), and different sample type (urine or kidney tissue):

1. The Higgins dataset [34] represents gene expression values from seven dissected compartments of the healthy adult human kidney, analysed by microarray technology.

2. The Chabardés-Garonne [35] dataset represents gene expression values from eight dissected compartments of the healthy adult human kidney, analysed by the Serial Analysis of Gene Expression (SAGE) method.

3. The EuReGene dataset [36] represents gene expression values from all the different renal structures of the healthy adult mouse kidney, analysed by the *in situ* hybridisation technique.

4. The Vlahou [37] and the Mann [38] datasets represents protein expression values from healthy adult human urine, analysed by different mass spectrometry techniques.

For each of these experiments the output is typically a list of genes or proteins of interest. The initial challenge is to identify proteins found in the urine and related them back to genes expressed in the kidney. This is made difficult as the data are coming from a range of experiments on both mice and humans. Background knowledge about genes and proteins is combined with experimental findings to generate new data for further analysis. The use of ontologies to annotate these data adds value, such as the ability to generalise over the observations. This combined data will provide input-data to a series of data-mining experiments within the e-LICO project.

**Figure 1:**
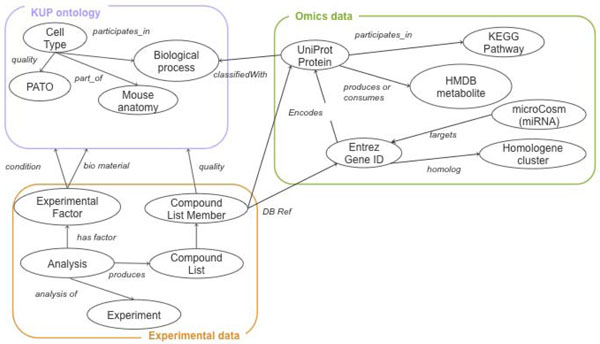
**Schema overview**. Overview of the KUP KB schema showing experimental data connected to background knowledge and annotated with the KUP ontology

We need the KUPKB to answer a series of queries about biological compounds in the urine and kidney. To achieve these we need basic information about the compounds under analysis. Information about genes, proteins and metabolites can be harvested from publicly available databases. These data must be linked to evidence coming from the experimental analysis and appropriately annotated to distinguish between the different experimental factors, such as the biological material or pathological state. To tie this data together we need ontologies that describe the relationships between the various datasets. These will include an ontology for the kidney and urinary pathway system, experimental analysis and biological databases. We use Semantic Web technologies to build the KUPKB so we can exploit the ontologies for querying and inference. RDF gives us language to represent the data plus a means to publish it on the Web following Linked Data principles [39]. Ontologies provide both the schema for the KUPKB and a controlled vocabulary for data annotation. We conceptually divide the KUPKB into three overlapping sets of ontologies (Figure 1). The first set of ontologies provides a domain vocabulary for describing the kidney and urinary pathway. This Kidney and Urinary Pathway Ontology (KUPO) describes the cells of the kidney in terms of their function and their anatomical locations. The second set describe the experimental data, this includes descriptions of the experimental results along with meta-data about the experiment, such as the experimental factors under observation. The final set of ontologies describe the data obtained from various external biological databases.

The life sciences are now rich in ontologies to support our task [22,40]. We can take advantage of these efforts and use fragments of these ontologies to build the KUPKB. Re-using existing ontologies in the KUPKB offers many advantages from an integration point-of-view: by adopting standards and exploiting existing annotation efforts [41] we have a greater potential for future integration of the KUPKB with other similar applications.

### Kidney and urinary pathway ontology development

The kidney enables the filtration of waste from the blood in the form of urine. Schematically, the kidney can be divided into four major compartments: 1) the glomerular compartment, involved in blood filtration, 2) the tubular compartment, involved in the fine tuning of urine composition, 3) the vascular compartment, involved in renal blood supply and 4) the interstitial compartment that surrounds the other structures. Each kidney compartment is formed from a wide variety of cell types, and the specificity of the compartments relies on these specialised cell functions. Depending on the aetiology, renal diseases may differentially affect the renal cells and the kidney compartments (e.g. diabetic nephropathy affects mainly the glomerular compartment whereas obstructive nephropathy affects mainly the tubular compartment). It is important to link back the disease processes to the anatomical alterations, as it will help not only to better understand the pathological mechanisms, but also to adapt therapeutic strategies. For these reasons, the first version of the KUPO imports ontologies to describe the anatomy, cell types and biological function associated with the cells of the kidney.

Reference ontologies for the components of the kidney and urinary pathways are readily available through resources such as OBO foundry [22] and BioPortal [40]. KUPO is a set of OWL classes that represent the cells of the kidney. The classes are described using logical definitions that use conceptualisations taken from external ontologies relevant to the KUP. This modular approach avoids repeating any previous development efforts, and focuses on extending and enriching pre-existing ontologies where necessary.

Two ontologies were initially considered to describe the anatomy of the kidney: the Foundational Model of Anatomy (FMA) [42] and the Mouse Adult Gross Anatomy Ontology (MAO) [43]. Domain experts inspecting the KUP portion of the FMA found that there was too much detail in some sections and not enough in others. In addition, too many ontological distinctions were made within this portion of the FMA and the consequent dispersal of information made it hard for our collaborating biologists to use. In time, we could have refined views of the FMA to do the job required, but we found that the MAO had all the detail for our needs. Furthermore, despite the *connecting tubule* being absent in mouse and present in humans, we still find this concept in the MAO. Therefore we concluded that the MAO can act as a substitute for the human anatomy, at least as far as the kidney is concerned.

This approach may be acceptable in the short term. There is, however, the issue that we effectively label human entities as being mouse entities. A solution would be a species neutral vertebrate anatomy, so any renal cell from any vertebrate species could be labeled as *renal cell*, and the species recorded elsewhere (see [44]. Efforts such as CARO (Common Anatomy Reference Ontology) [45] are attempting to provide cross species reference ontologies for anatomy. The Vertebrate Bridging Ontology (VBO) project [46] has recently started and we will look to use such efforts in the future.

The Cell Type Ontology (CTO) [47] was the obvious choice for cells, but was found to be lacking a large number of known renal cell types. A list of new cells was therefore generated and cells were described in terms of their anatomical location using the *part_of* relationships from the Relations Ontology (RO) [48]. By exploiting the rich partonomy of the MAO we could use the transitive characteristic of the RO *part_of* relationship to describe the renal cells using equivalent class axioms. The logical definition meant that the complete classification of renal cell types could be computed using an OWL reasoner. The renal cells were further described in terms of the biological processes from the Gene Ontology (GO) [49] in which they participate. These new cell types are to be submitted for inclusion into the CTO.

### KUPO development

Populating the KUPO requires detailed domain knowledge about the anatomy and cells of the kidney. We wanted to explore methodologies that engaged the domain expert in the ontology building process. Modern ontology editors, such as Protégé [50], are powerful, but necessitate a relatively steep learning curve that can be dissuasive for domain-experts. We attempted to construct the ontology using simple templates that could be populated by domain experts to gather knowledge about the kidney. Populated templates were then converted to OWL to produce the KUP ontology.

**Figure 2:**
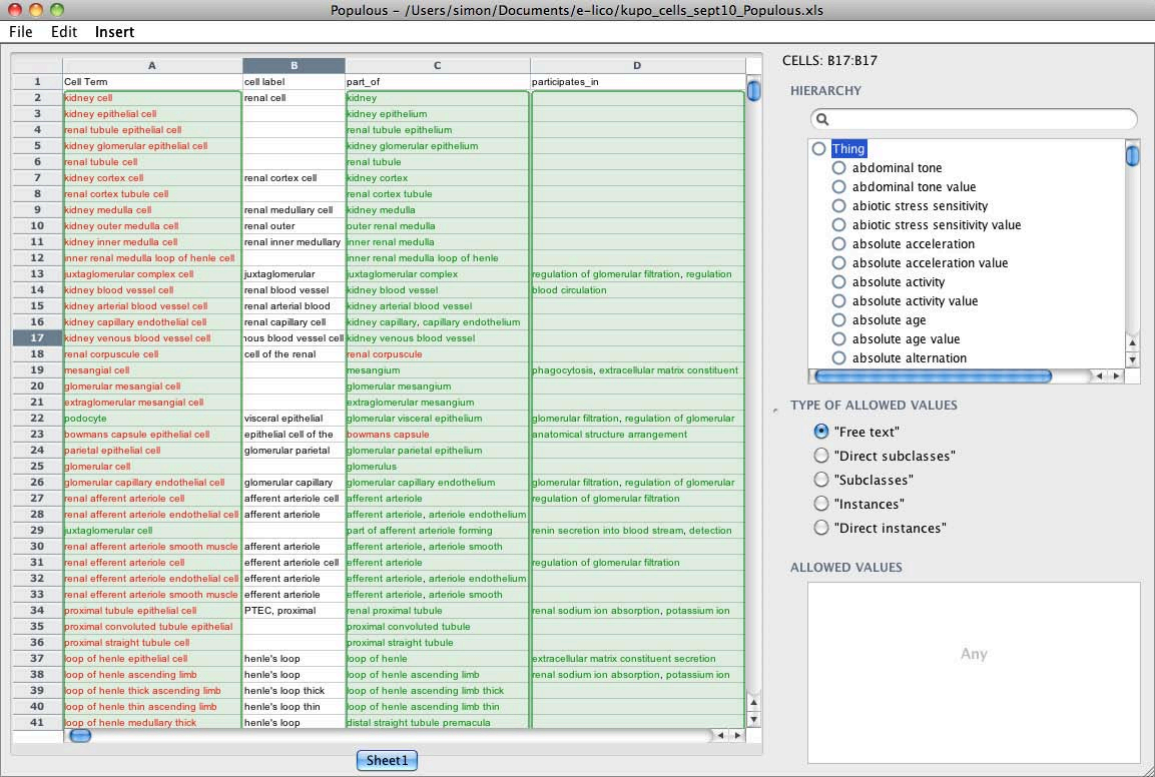
**Populous.** Screenshot of Populous showing template population of the KUP ontology

The templates were generated using a simple spreadsheet, a technique that has recently become popular in other ontology efforts [51,52]. A KUPO day was also held as part of a meeting between domain experts from the EuroKUP consortium [53] to develop and review the knowledge captured in the spreadsheet. These spreadsheets were validated and transformed into OWL using the Populous tool [54]. Figure 2 shows the KUPO template populated in Populous. A detailed description of Populous and the KUPO development methodology is available in [54].

### Background knowledge

Background knowledge in the KUPKB is composed of various external databases represented in RDF. Converting existing data into RDF triples is relatively straight forward. Our methodology closely mimics the development of the HCLS knowledge base [55]. Additionally the Bio2RDF project [32] provides a repository of public databases that can be downloaded in RDF. These two efforts provided some of the core datasets for the background biological knowledge represented in the KUPKB.

The initial KUP experiments are concerned with genes and proteins expressed in kidney and urine samples, while future datasets will be generated from metabolomic and microRNA studies. We selected the Entrez Gene database [56] for gene annotations and UniProt Knowledgebase [57] for proteins, including the Uniprot Gene Ontology annotations [41]. KEGG [58] provides the data for biochemical pathways whilst the microCosm database [59] provides microRNA target prediction sites. Given that the experiments are being conducted on multiple species, we also required data about orthologous genes that can be obtained from the Homologene database [60].

We wished to capture the relationships between the various entries in these databases to form a large connected graph of data. Figure 1 gives an outline of how our background knowledge relates to the KUPO and the experiments. Where possible we used the Bio2RDF ontology [61] to provide a simple schema for the different databases.

RDF representations of Entrez Gene, Uniprot, Homologene and KEGG can be obtained from Bio2RDF. Uniprot KB also provide periodic releases of their database in RDF. Whilst it was possible take the complete databases and place them directly into the KUPKB; it was neither necessary or desirable to replicate these databases in their entirety. Instead we reuse URIs from Bio2RDF and Uniprot KB to reference external entities in the KUPKB. By re-using URIs from external resources we can expose the KUPKB as *Linked Data* and harvest additional data if and when we need it. Resources in the KUPKB have URIs that resolves to documents on the Web; these documents provide a description of that resource in RDF and link directly to other documents that describe the external resources using RDF. This linking connects the KUPKB into the growing web of linked life science data and highlights one of the major advantages of a Semantic Web approach.

### KUPKB experiments ontology

The KUPKB is required to capture findings from biological experiments such as lists of interesting genes or proteins expressed under certain conditions. Ontologies such as OBI [62] and EFO [63] provide concepts for the annotation of design, protocols, instrumentation, materials, experimental factors and data associated with a biomedical investigation. Despite the extensive coverage of these ontologies, neither provided a clear way to represent information in gene and protein lists. It is particularly difficult to attach the appropriate meta-data to these lists in a way that could accommodate a wide variety of use-cases.

It is beyond the scope of this work to provide a complete modelling solution to this problem, and work is underway through projects like OBI and the BioRDF task force of the HCLS working group to create standard modelling patterns for this kind of data. We opted to build our own ontology to model this data that was simply driven by requirements of the queries we needed to ask. Though, with a view to future integration with external ontologies, we followed patterns and reused concepts from existing ontologies, including OBI, EFO and PATO [64].

Each experiment in the KUPKB is modelled as an instance of a particular ‘*experimental assay type’*. Each *‘experimental assay type’* can have multiple *‘experimental analysis’* associated with it. Each *‘experimental analysis’* is annotated with an *‘experimental factor’* and produces some *‘data’*.

**Figure 3:**
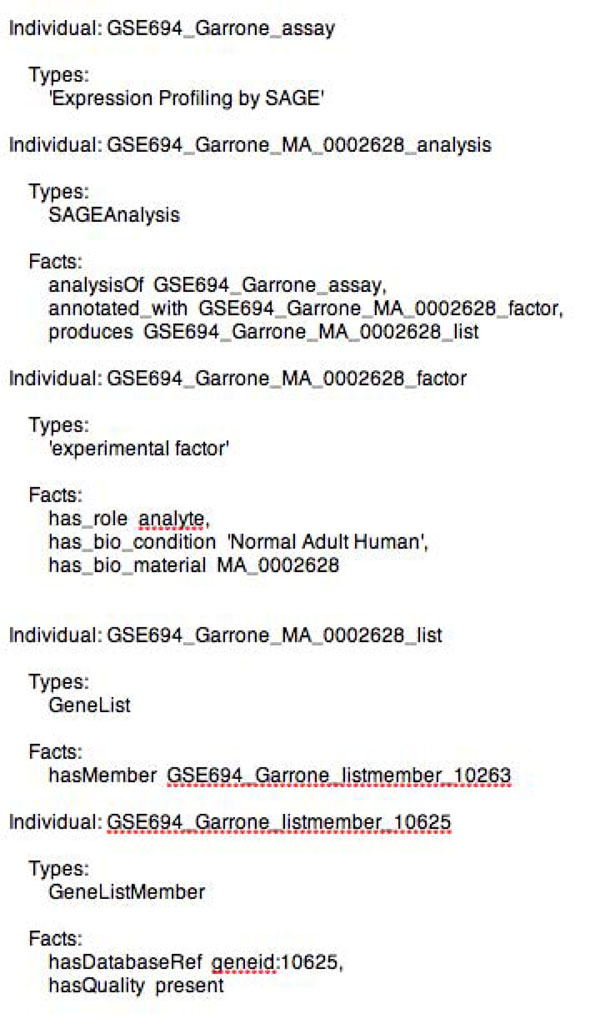
**Gene lists in OWL.** Manchester OWL syntax for the asserted information representing a single analysis of the Garonne dataset

For example, the Chabardés-Garonne dataset [35] represents gene expression values from eight dissected compartments of the healthy adult human kidney. This experiment is described as a type of *‘Expression Profiling by SAGE’*. We create instances of *‘experiment analysis’* to represent each of the factors under investigation. In this case, the analysis represents gene expression sets for dissected compartments of the kidney, as published by the authors. *‘Experimental factors’* can have a related sample (e.g. glomerulus), a related condition (e.g. *‘Adult Human’*), and an associated role in the experiment (e.g. *‘control’ or ‘analyte’*). The resulting *‘gene list’* for a particular analysis is related to multiple *“gene list members’*. Each member in a gene lists can be annotated with additional meta-data such as an external database reference (e.g. Entrez Gene id for genes). We have adopted terminology from PATO to describe attributes for the *‘gene list members*’ such as *‘postitive regulation’* or *‘present’*. Figure 3 is an OWL representation of a single gene in the gene list from the Garonne dataset. We make no claims about our choice of modelling other than it is sufficient for the kinds of queries we need to ask over the current set of proposed experiments in the KUPKB.

### KUPKB architecture

There are now many options for developers wishing to deploy RDF and OWL data, the most common approach to date is the use of a triple store. Triple stores provide a framework for storing and querying RDF data, along with support for varying degrees of inferencing. One of the major factors in selecting a triple store is scalability. In recent years triple stores have improve to accommodate large amounts of RDF data (billions of triples). See here [65,66] for a review of some popular triple stores. We opted to deploy the KUPKB data using Sesame [67] backed with a storage and inference layer provided by BigOWLIM [68]. Sesame provides a powerful yet simple framework for managing and interfacing with the RDF data whilst BigOWLIM provides a fast and scalable implementation of the Sesame Storage and Inference Layer (SAIL). In order to expose the KUPKB data as Linked Data, we use the Pubby [69] service. A simple description of every resource in the KUPKB can be retrieved by its URI as either a human readable HTML document or plain RDF.

## Results

The KUPKB can be queried over the Web via a SPARQL endpoint [70]. The KUPKB Web page provides some example SPARQL queries to generate interest and harvest requirements from the wider KUP community. The demo shows how one can query across multiple data sources using predicates and terminology from the available ontologies. At the time of writing, the total number of RDF triples in the KUPKB is 10,415,339, whilst the ontologies and experiments describe 24,557 classes and 10,539 individuals. To date no triple stores support the full expressivity of OWL-DL or OWL 2; in order to compensate for this we attempted to compute any inferred knowledge before loading the data into the knowledge base. We attempted to classify the complete knowledge base with three OWL reasoners; Pellet 2.1.2 [71], Fact++ 1.5.0 [72] and HerMiT 1.3.1 [73] on a 2 x 2.66GHz Dual Core Intel Xeon Mac Pro with 16GB or RAM and running OS X server 10.6.4. No reasoner was able to classify the complete knowledge base in our experiment, however, by excluding the large external databases we were able to classify the various ontologies along with the experimental data in reasonable time (4.30 min with Fact++). This classification step provided a level of consistency checking and also enabled the computation of missing subsumptions to form the KUPO class hierarchy.

The first set of demo queries answer simple questions from the KUPO. For example, ‘Which biological processes occur in the kidney collecting duct?’ is answered by getting all the cells from the CTO and the KUPO that participate in a biological process from GO, and filtering these results on cells that are part of the collecting duct from MAO.

More interestingly, the KUPKB demonstrates how having access to experimental observations along-side existing domain knowledge could be useful for hypothesis generation and experimental evaluation. The query ‘Which genes have evidence for upregulation the glomerulus of a normal adult kidney?’ can now be answered by getting all genes annotated with *‘upregulated’* from all experiments where the condition is *‘normal adult human’* and the bio material is *‘glomerulus’*. This kind of query demonstrates the added value KUPO brings. Not only do we get answers for genes that are expressed in the glomerulus, but by exploiting the transitive nature of the *part_of* relationship, we infer genes upregulated in experiments on any part of the glomerulus. We can extend this query by exploiting the KUPO to ask for all the cells that are in parts of the kidney expressing protein involved in some biological process or pathway.

Our final query demonstrates how we can begin to answer real questions from the KUP scientists. The initial goal was to place protein expressed in urine back into the context of the kidney. The Vlahou and Mann datasets [37,38] both provide us with sets of protein identified in urine from normal adult humans. We want to compare these results with evidence for gene expression in particular compartments of the kidney. Our example query gets all the proteins found in urine across both experiments. We need to find the intersection of proteins in this list with proteins expressed in both the Higgins [34] and Garonne [35] datasets. To do this we must first map the genes to their respective protein using background knowledge in the KUPKB. We can get the Uniprot identifiers for each gene expressed in the transcriptomics datasets. By combining these two sets of derived protein we find that 183 proteins have evidence for expression in seven separate compartments of the kidney. We can further enrich this list with their appropriate GO annotations to see if there are any patterns observed in this list. We can now extend this query using data from homologene to bring in the EuReGene dataset from mice to collect further evidence.

The flexibility offered by KUPKB provides the e-LICO project with a platform to plan data mining experiments over the KUP data. A recent development from e-LICO was the initiation of a KUP challenge; this is a challenge for the data mining community to learn models from datasets relating to Obstructive Nephropathy (ON) in children [74]. These datasets are of high dimensionality, but extremely small sample size. The datasets represent analysis from multiple biological levels including miRNA, mRNA, proteomics and metabolomics; the challenge is to build a prediction model from these datasets that uses background knowledge in the KUPKB to connect the different levels.

## Discussion

We have presented an approach to developing a bespoke knowledge base for integrating biological data relating to the KUP. This KUPKB integrates experimental findings with background knowledge to provide input for data mining experiments on the e-LICO platform. The KUPKB differs from more traditional database approaches by its extensive use of Ontologies and Semantic Web technologies to provide the underlying data model. The KUPKB is an example of the levels of data integration that can be achieved once data can be reduced to a common language. Having access to multiple connected data sources in a single RDF repository provides a powerful and flexible platform for data gathering and analysis. The additional semantics provided by the ontologies offer new and more flexible ways to query and explore the data. Despite the potential, there are, however, many outstanding issues that continue to stifle development in this area.

We found that taking an approach using RDF to gather our data in one form worked well. As well as taking advantage of existing resources in RDF, conversion of bespoke KUP experimental data was relatively straight forward using simple scripts. The main difficulty was reconciling identifiers across resources; for each experimental dataset we encountered, a considerable effort was required to map the various identification schemes between databases. Whilst this was to be expected for the biological databases, it was a surprise to see that little authority exists within the life sciences on the correct URIs for various ontologies and RDF datasets. For example, we encountered multiple URIs for common predicates such as the *part_of* relationship from the OBO relations ontology.

The KUPKB still has aspects of a warehouse; it gathers resources together in one place in one form. The KUP ontology is used as a schema for the data. Some of the dangers of warehouses are, however, avoided. The Semantic Web technologies are tolerant, indeed suited for, irregular and incomplete data. Production of data in the common form of RDF is distributed and much of the task of forming the KUPKB is the ‘gathering’. The emerging ontological *lingua franca* makes mapping to a schema relatively straight-forward. An early requirement for the KUPKB was an ontology that accurately described the kidney and urinary system. Given the widespread availability of anatomical ontologies that describe the renal system, we decided to focus on the cells of the kidney, that previously had little coverage in existing ontologies. The challenge was to engage the domain experts in the development of such an ontology. We managed to achieve this by shielding the experts from the underlying ontology building, and instead gathered knowledge using a simple template approach. The templates were populated in spreadsheets, an application familiar to the domain scientists. This approach led to the description of over 180 renal and urinary cell types by domain experts with little or no ontology building experience.

In order to support the validation of these spreadsheets and their transformation into an ontology, we developed an application called Populous [54]. Populous enables us to separate the axiom pattern in the ontology from its population. The pattern itself was simple, but in a pattern with few axioms and 180 repetitions, we generated an ontology with several hundred asserted and inferred axioms. The Populous approach allows this generation to happen quickly and with utter consistency. If the pattern changes, but with the knowledge the same, then update is equally quick. Any change to the knowledge can be done in the table environment that validates input against constraints.

This first version of KUPO serves its purpose as an application ontology. Questions, however, still remain about our choice of modelling for this ontology. We used *participates_in* to relate a cell to the process in which it is involved, even though we know this is not necessarily true for all cells of a given type at all times. We could model that these cells have a disposition that is realised in the biological process, but this kind of distinction does not add any value to the kinds of queries we want to ask. We have deliberately moved away from a representation of the“truth” of the biology in order to accommodate the queries our users wish to make. Problems of “truth” continue to plague us in the development of an ontology to describe our experiments.

Developing a common ontology for biomedical experiments and findings is difficult as the semantics of a biological finding often involve a complex set of interactions. If we take our gene lists in the KUPKB as an example; these represent some experimental finding that a biological entity is either present or absent under a certain condition. The validity of such a statement should take into account a host of factors including the type of experiment, which instruments were used, how the data was analysed, along with consideration for less controllable factors such as human error or bias. The development of appropriate ontologies that capture these various attributes are well under way, however, progress is inevitably slow due to the complex nature of the task. For the KUPKB we initially have few requirements for complex descriptions of the experiments. Datasets are being selected by a relatively small community, and much of this data is similar enough for cross-comparison. As the KUPKB expands to include more heterogeneous datasets, more accurate description of the data may well be required in order to ask more complex queries of the data. Developments in this area is ongoing and we will contribute experiences from this exercise to the appropriate ontology efforts. We believe that a relatively simple ontology, similar to our experiments ontology, could provide a model that is “just enough” to achieve more wide spread integration of this kind of data.

One of the attractive features of adopting ontologies and technologies like OWL is the ability to exploit the semantics of the language to check for consistency and draw inferences from the data. Where possible we exploited OWL semantics to drive inferences and reduce the number of manual assertions we needed to make in our ontology. This has many advantages from an ontology maintenance point of view and provides us with with advanced querying capabilities, such as asking for parts of the renal cortex and returning all the parts right down to the cells. The ability to traverse transitive relationships is one of the benefits an ontology language can bring; it enables us to ask general queries such as asking for all cells that participate in cytokine production, and by inference would also return cells that participate in specialisations of cytokine production, such as B cell cytokine production.

Despite the expressive power of OWL queries, we hit a limit when we try to populate these ontologies with instance data; current OWL reasoners do not easily scale to the volumes of data presented in the KUPKB, which only represents a very small dataset in comparison to other efforts such as the Alzeimers knowledge base. As a practical solution to this problem we use an RDF database, which can scale to many billions of triples, and exploit the limited, but adequate, inferencing capabilities currently on offer.

By moving to an RDF store we lose the ability to ask queries that use higher level OWL constructs. An RDF query language like SPARQL enables us to explore the structure of an OWL ontology, but queries soon become complex when working with class level descriptions. Our chosen RDF store BigOWLIM provides inferencing up to the level of RDFS along with a fragment of OWL that can be expressed using rules, such as transitivity and *same as*. We take the attitude that some answers (that may be incomplete) are better than no answers from a sound and complete query solution. The reduction in expressivity within the RDF domain means that the queries asked may not be as precise; to date we have not yet found this to cause significant problems, though this may not be the case in the future. Work to increase the expressivity of SPARQL with respect to OWL semantics is underway through current proposals such as SPARQL 1.1 entailment regimes [75,76] and SPARQL-DL [77].

The technical limitations associated with querying a knowledge base like the KUPKB has an impact on how we build our ontologies and model our data. Most ontologies for the life science model biological phenomena at the class level. For example, the class *‘cytokine production’* describes all instances of the process of cytokine production. If we treat a particular protein record from Uniprot as an instance, then to represent the relationship in OWL we would type this instance as a member of the class of things that participate in cytokine production. Representing OWL axioms such as this in RDF soon becomes verbose and impacts on the complexity of the query. In order to simplify the query we treat the classes as individuals and create a binary relationship between the two.

### Future work

The KUPKB is to be extended with several datasets from experiments about specific types of renal disease. These include proteomic analysis of urine from children suffering obstructive nephropathy. The KUP ontology will be extended to incorporate the appropriate ontologies that describe the diseases under investigation. These data will serve as input to data mining experiments within the e-LICO project, with the ultimate goal of generating new predictive models for renal disease. Early experiments have shown that we can find useful and interesting correlations in the data pulled from the KUPKB.

A further issue to tackle is the update of resources. Experiments change; new data emerge; the ontologies describing these resources change. Keeping resources like the KUPKB up-to-date will be a struggle. Tools such as Populous will help with re-generating the ontology, but the mappings to the ontology all have to be managed, exposed and recorded. In addition, the provenance of the input from all the resources needs to be recorded to aid scrutiny.

At present we have not recorded much information about the experimental protocols. Currently, the experiments we include in the KUPKB have been chosen by our participating biologists. In future we will need to describe the experiments such that scientists can have access to a wide variety of experiments and make the choice themselves. This final point of scientists interacting with the KUPKB highlights the need for good interfaces for querying and browsing such knowledge bases.

## Conclusion

We have presented an approach to developing a knowledge base to serve a community of scientists working on kidney and urinary pathway diseases. We have demonstrated how a knowledge base such as this can be rapidly developed using state-of-the-art SW tools. In the KUPKB we have taken advantage of the *de facto* integration described as an aim of ontologies such as GO [78], by using the ontologies themselves along with data annotated with those ontologies to provide an integrated resource of data about KUP for a community of biologists. The KUPKB can provide useful querying facilities to biologists and has been relatively easy to produce. There remains much to do, but we count the work so far as a success.

## Competing interests

The authors declare that they have no competing interests.
